# Uncommon Presentations of Endometriosis: Clinicopathological Features of Abdominal Wall and Extrapelvic Lesions

**DOI:** 10.3390/jcm15103889

**Published:** 2026-05-18

**Authors:** Ismet Hortu, Mert Acar, Cagdas Sahin, Ali Akdemir, Levent Akman, Fatih Sendag, Murat Ulukus

**Affiliations:** 1 Department of Obstetrics and Gynecology, Faculty of Medicine, Ege University, Izmir 35100, Turkey; cagdas.sahin@ege.edu.tr (C.S.); ali.akdemir@ege.edu.tr (A.A.); levent.akman@ege.edu.tr (L.A.); fatih.sendag@ege.edu.tr (F.S.); murat.ulukus@ege.edu.tr (M.U.); 2 Department of Obstetrics and Gynecology, Nusaybin State Hospital, Mardin 47300, Turkey; drmertacar@gmail.com

**Keywords:** endometriosis, abdominal wall, cesarean section, gynecologic surgical procedures

## Abstract

**Background/Objectives**: Abdominal wall and extrapelvic endometriosis are uncommon entities that may mimic other surgical conditions and delay diagnosis. This study evaluated their clinicopathological, diagnostic, and surgical features in a single-center case series. **Methods**: This retrospective study included 29 patients with histopathologically confirmed abdominal wall or extrapelvic endometriosis treated at a tertiary referral center between 2009 and 2025. Demographic and clinical characteristics, surgical history, CA-125 levels, imaging findings, lesion size, and surgical features were analyzed. Abdominal wall cases were further evaluated based on the presence of muscle or fascial invasion. **Results**: Abdominal wall lesions comprised 93.1% of cases, while extrapelvic lesions (6.9%) were all vaginal. Most cases had a history of cesarean section; however, one patient had no prior abdominal surgery, consistent with spontaneous disease, with concomitant endometrioma and deep infiltrating endometriosis. Muscle or fascial invasion was observed in 63.0% of cases. Both CA-125 levels (*p* = 0.005) and CA-125 positivity (≥35 U/mL) (*p* = 0.029) were significantly higher in patients with invasion. Cyclic symptoms were present in 89.7% of patients, and mesh repair was required in two cases with large lesions. **Conclusions**: Abdominal wall endometriosis should be suspected in patients with cyclic pain or swelling at surgical sites, particularly after cesarean delivery, although it may occur without prior surgery. Deep muscle and fascial invasion may be associated with elevated CA-125 levels and increased CA-125 positivity, sometimes requiring wider excision and mesh repair. These findings may support earlier diagnosis and surgical planning.

## 1. Introduction

Endometriosis is a common benign gynecological condition characterized by the ectopic presence of endometrial glands and stroma, which induces a chronic inflammatory reaction. It affects approximately 10% of women worldwide, with a higher prevalence among those with infertility. Clinical manifestations include chronic pelvic pain, dysmenorrhea, dyspareunia, abnormal uterine bleeding, and infertility [1].

Beyond its traditional definition as a pelvic gynecological disorder, endometriosis is increasingly recognized as a chronic systemic inflammatory condition with complex biological and clinical consequences [2]. This broader understanding is supported by evidence demonstrating associations with multiple comorbidities, including autoimmune, metabolic, and cardiovascular diseases. Accordingly, a narrow focus limited to reproductive symptoms may fail to capture the full burden of the disease [3]. In addition to these associations, endometriosis has been linked to a wide spectrum of clinical manifestations beyond the pelvis, further supporting its systemic nature. The underlying mechanisms are thought to involve dysregulated immune responses, chronic inflammation, and altered hormonal signaling pathways, which may contribute to both local lesion development and systemic effects [4].

Moreover, the heterogeneity of clinical presentations may lead to diagnostic delays, particularly in cases with atypical or extrapelvic manifestations. Therefore, increased clinical awareness and a more comprehensive diagnostic approach are essential to avoid underdiagnosis. In this context, management strategies focusing solely on gynecological aspects may be insufficient, and a multidisciplinary approach integrating gynecological, surgical, and other relevant specialties may provide more effective patient care [5].

From a therapeutic perspective, treatment strategies targeting only the reproductive tract may be insufficient to address the systemic burden of the disease. Surgical excision remains the primary treatment modality for most extrapelvic lesions, particularly in abdominal wall endometriosis, where complete resection is associated with low recurrence rates. In selected cases, adjunctive hormonal therapy may be considered to reduce recurrence risk, although its role in extrapelvic disease remains less clearly defined. Given the complexity and heterogeneity of the disease, a multidisciplinary approach integrating gynecological, surgical, radiological, and other relevant specialties may provide more effective patient care [6].

Extrapelvic endometriosis refers to the presence of endometrial tissue outside the pelvic cavity and may involve locations such as the cervix, vagina, abdominal wall, gastrointestinal tract, urinary tract, diaphragm, and thorax [7]. Among these, abdominal wall endometriosis (AWE) is one of the most frequently reported forms of extrapelvic disease and is strongly associated with previous gynecologic or obstetric surgery, particularly cesarean section. However, although rare, cases of abdominal wall endometriosis occurring in the absence of prior cesarean section or other abdominal surgery have been previously reported in the literature [8]. Vaginal endometriosis is less common and is usually considered a form of deep infiltrating endometriosis, although involvement of the distal vagina has been rarely reported [9].

Patients with abdominal wall endometriosis typically present with cyclic pain, a palpable mass, or swelling at the site of a previous surgical scar [10]. Imaging modalities such as ultrasonography, computed tomography, and magnetic resonance imaging may assist in diagnosis, but definitive diagnosis requires histopathological confirmation after surgical excision. In some cases, the lesion may extend into the rectus muscle or fascia, requiring more extensive surgical resection. Reconstruction with mesh may be necessary when large defects occur after excision [11]. Serum cancer antigen-125 (CA-125) has been investigated as a potential marker in endometriosis, but its clinical significance in abdominal wall or extrapelvic lesions remains unclear.

Because abdominal wall and extrapelvic endometriosis are relatively rare conditions, available data regarding clinicopathological characteristics, depth of invasion, and surgical management are limited. The aim of this study was to evaluate the clinicopathological features, diagnostic findings, and surgical characteristics of abdominal wall and extrapelvic endometriosis in a single-center case series and to analyze factors associated with muscle and fascial invasion.

## 2. Materials and Methods

### 2.1. Study Population

**Figure 1:**
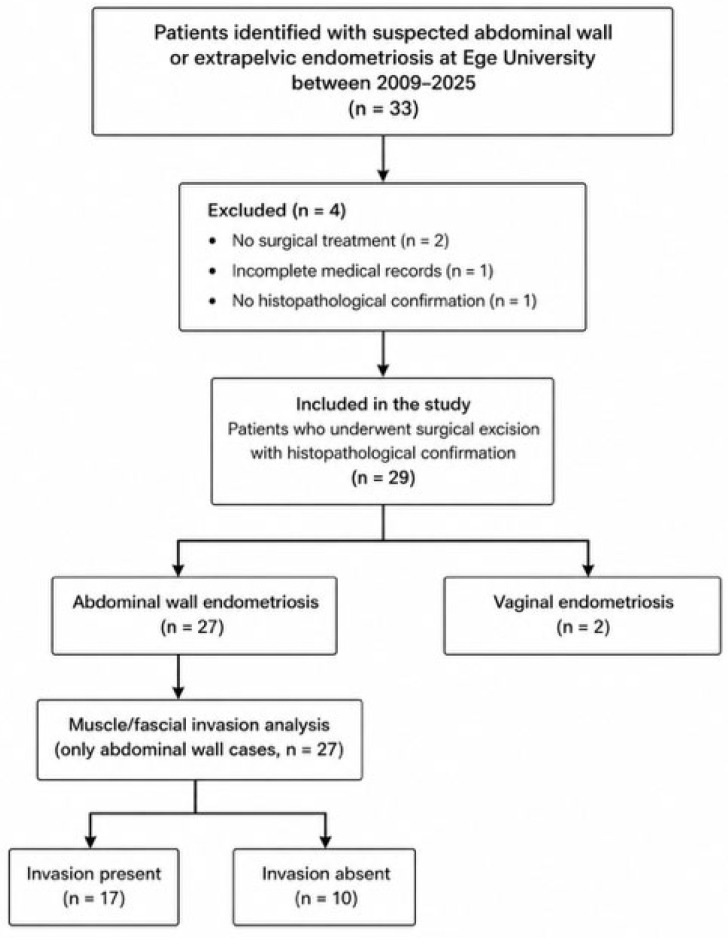
Flowchart of patient selection, exclusion criteria, and subgroup classification.

This retrospective study included 29 patients diagnosed with abdominal wall or extrapelvic endometriosis at the Department of Obstetrics and Gynecology, Ege University Faculty of Medicine, between 2009 and 2025. All patients underwent complete surgical excision of the lesion, and the diagnosis of endometriosis was confirmed histopathologically. Patients were included if they had undergone surgical excision of the lesion with histopathological confirmation of endometriosis. Patients without surgical treatment, without histopathological confirmation, or with incomplete medical records were excluded. Patients were retrospectively identified based on surgical and histopathological records. A flowchart illustrating the patient selection process is provided in Figure 1.

### 2.2. Data Collection

Medical records were reviewed to obtain demographic characteristics, clinical symptoms, history of previous abdominal surgery, imaging findings, serum CA-125 levels, lesion size, lesion localization, and operative findings. Particular attention was given to the presence of muscle or fascial invasion in abdominal wall lesions. In addition, the need for mesh repair after excision was recorded. Abdominal wall cases were further analyzed according to the presence or absence of muscle/fascial invasion.

Clinical and histopathological data were retrospectively extracted from electronic medical records by the study investigators using predefined variables. Uniform definitions were applied, and the same data extraction approach was used for all patients to ensure consistency. Serum CA-125 levels were measured in the same institutional laboratory using standardized methods, ensuring comparability across patients. Given the retrospective design, blinding was not feasible.

### 2.3. Ethical Approval

The study protocol was approved by the Ege University Faculty of Medicine Medical Research Ethics Committee (approval number: 23-5T/43). The study was conducted in accordance with the principles of the Declaration of Helsinki.

### 2.4. Statistical Analysis

Statistical analysis was performed using IBM SPSS Statistics version 27.0 (IBM Corp., Armonk, NY, USA). Continuous variables were expressed as median (interquartile range [IQR]), and categorical variables were expressed as number (percentage). The Mann–Whitney U test was used for a comparison of continuous variables between groups. The chi-square test or Fisher’s exact test was used for categorical variables, as appropriate. A *p* value < 0.05 was considered statistically significant.

## 3. Results

### 3.1. Patient Characteristics

The clinical and demographic characteristics of the patients are summarized in Table 1. A total of 29 patients with histopathologically confirmed abdominal wall or extrapelvic endometriosis were included. The median age at diagnosis was 36 years (IQR: 9), and the median body mass index (BMI) was 27.1 kg/m^2^ (IQR: 5.4). The median parity was 2 (IQR: 1). Abdominal wall endometriosis accounted for 93.1% of cases, whereas extrapelvic endometriosis was observed in 6.9%, all of which were located in the vaginal region. The majority of abdominal wall endometriosis cases had a history of previous cesarean section; however, one patient had no history of prior abdominal surgery. Previous non-cesarean abdominal surgery was present in 34.5% of patients. Elevated serum CA-125 levels (≥35 U/mL) were detected in 69.0% of cases, with a median value of 40 U/mL (IQR: 24). Concomitant endometrioma was present in 10.3% of patients. The median lesion size was 3 cm (IQR: 1.5). Symptoms related to the menstrual cycle, including cyclic pain or swelling, were reported by 89.7% of patients.

Among the abdominal wall endometriosis cases, muscle or fascial invasion was detected in 63.0% of patients. Mesh repair after surgical excision was required in two cases with large lesions (5 and 6 cm), both showing muscle and fascial invasion. Comparison of abdominal wall endometriosis cases according to the presence of muscle/fascial invasion is presented in Table 2. Patients with invasion had significantly higher CA-125 levels (*p* = 0.005) and a higher rate of CA-125 positivity (*p* = 0.029) compared with those without invasion. No significant differences were found in age, BMI, lesion size, or other clinical variables.

Additional subgroup analyses exploring associations between clinical variables are provided in Appendix A (Table A1). Patients with elevated CA-125 levels were significantly older. Higher BMI was associated with a greater number of previous cesarean sections and a higher frequency of ≥2 cesarean deliveries.

### 3.2. Imaging Modalities

**Figure 2:**
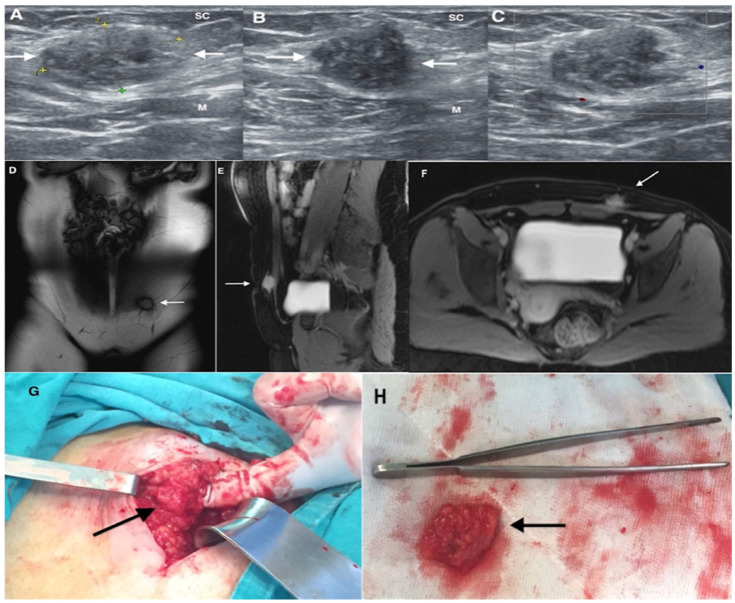
Abdominal wall endometriosis in a 28-year-old woman with a history of one cesarean section presenting with a palpable swelling and cyclic pain at the cesarean scar. (**A**) Transverse transabdominal ultrasound image shows a hypoechogenic, nodular mass (white arrows) with heterogeneous appearance and ill-defined borders, situated between the subcutaneous tissue (SC) and the rectus abdominis muscle (M). Colored calipers and numbered markers indicate the lesion margins. (**B**) Longitudinal transabdominal ultrasound image demonstrates the same lesion characteristics. (**C**) Color Doppler image reveals no vascularity within the lesion; (**D**) Coronal T2-weighted MRI depicts the lesion at the left corner of the cesarean scar. (**E**) Sagittal and (**F**) axial T1-weighted MRI with fat suppression demonstrate high lesion intensity relative to muscle. White arrows in (**D**–**F**) indicate the lesion. (**G**) Wide resection of the endometriotic lesion (black arrow). (**H**) Macroscopic view of the excised lesion (black arrow).

**Figure 3:**
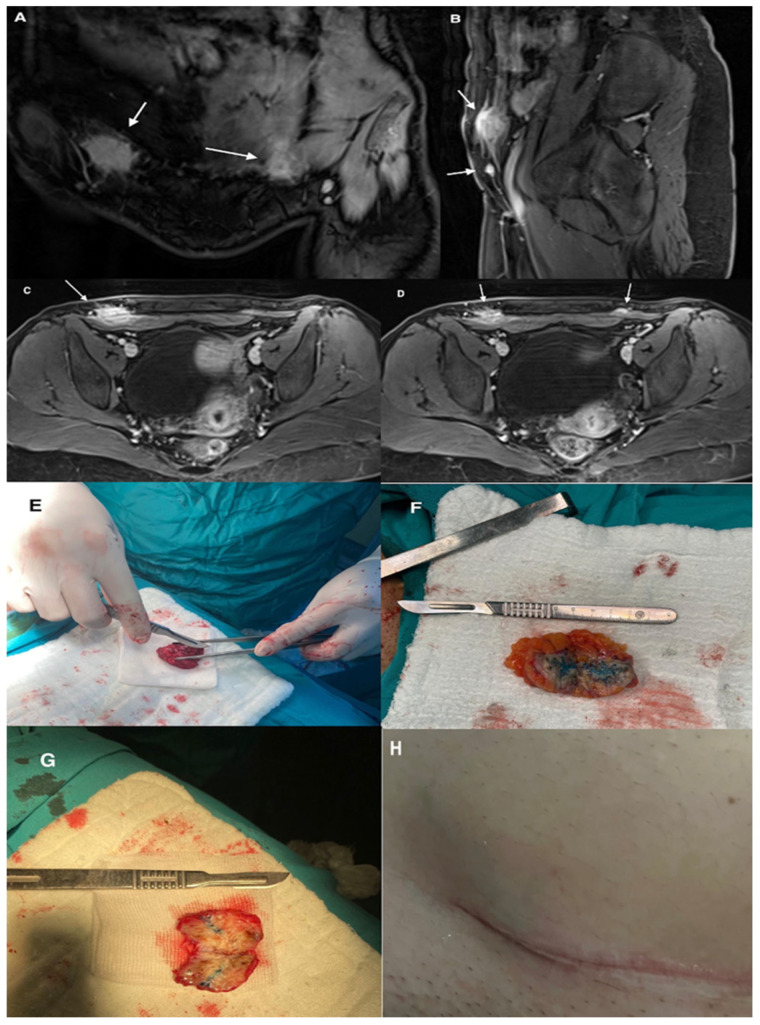
Bilateral abdominal wall endometriosis in a 30-year-old woman with a history of two cesarean sections, presenting with palpable swelling, discoloration along the cesarean scar line, cyclical pain, and abdominal and pelvic pain. (**A**) Coronal T1-weighted MRI illustrates bilateral lesions (white arrows). (**B**) Sagittal and (**C**,**D**) axial T1-weighted MRI with fat suppression demonstrate hypervascular and high-intensity lesions relative to muscle. White arrows in (**A**–**D**) indicate the lesions. (**E**–**G**) Methylene blue injected into the lesions during surgery highlights the central portions, aiding dissection. (**H**) Skin discoloration over the subcutaneous lesion on the right lateral side of the cesarean scar.

**Figure 4:**
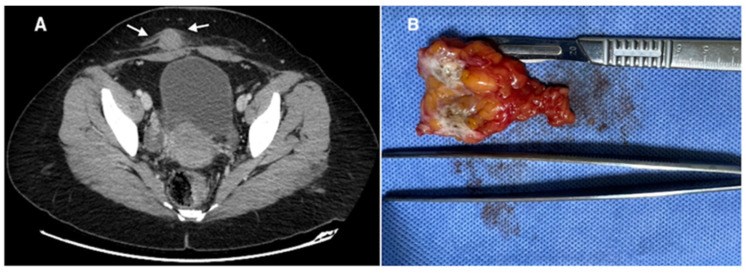
Abdominal wall endometriosis in a 34-year-old woman with a history of two cesarean sections, endometrioma excision, inguinal hernia repair, and appendectomy, presenting with cyclical pain, dysmenorrhea, and palpable swelling along a Pfannenstiel incision scar. (**A**) Axial computed tomography (portal venous phase) image reveals linear perilesional infiltration (white arrows); (**B**) macroscopic appearance of the excised endometriotic nodule.

Diagnostic imaging methods included ultrasonography, magnetic resonance imaging, and computed tomography. Imaging techniques were not uniformly performed in all patients. Radiologic evaluation was used to determine lesion size, localization, and depth of invasion before surgery. Representative imaging findings are shown in Figure 2, Figure 3 and Figure 4.

### 3.3. Histopathological Diagnosis

**Figure 5:**
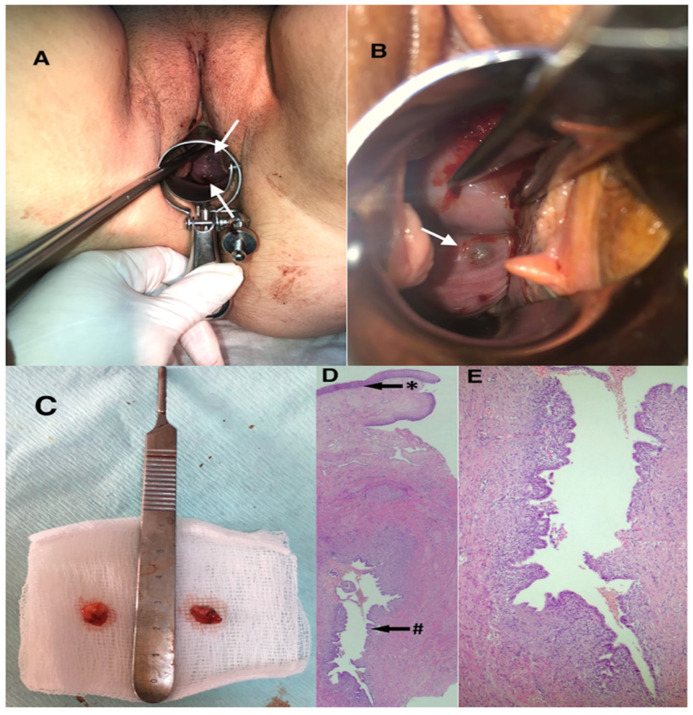
Vaginal endometriosis in a 33-year-old woman with two vaginal deliveries, presenting with abnormal uterine bleeding, pelvic pain, and dysmenorrhea. (**A**,**B**) Speculum examination revealed two endometriotic nodules (white arrows), measuring approximately 1 cm and 7 mm, extending from the cervix’s 6 o’clock position to the posterior fornix. (**C**) Macroscopic appearance of the excised lesions. (**D**) Hematoxylin and eosin (H&E) photomicrograph (20×): * squamous epithelium; # endometriosis cyst located in the deep dermis (black arrows). (**E**) H&E photomicrograph (100×): higher magnification showing endometrial surface epithelium lining the cyst’s inner surface and the underlying endometrial stroma.

Histopathological examination confirmed the diagnosis of endometriosis in all cases following complete surgical excision. Microscopic evaluation demonstrated endometrial glands surrounded by endometrial stroma. In some specimens, hemosiderin deposition within macrophages or stromal tissue was observed, consistent with repeated hemorrhage. Representative histopathological findings are shown in Figure 5.

## 4. Discussion

Abdominal wall endometriosis is an uncommon form of extrapelvic endometriosis and is still easily overlooked in daily practice. Although endometriosis primarily affects pelvic organs, extrapelvic disease accounts for a minority of cases, and abdominal wall involvement may be missed when clinical suspicion is low. Contemporary reviews describe abdominal wall endometriosis as a rare but increasingly recognized entity, particularly in association with prior abdominal surgery, most commonly cesarean section [12,13]. In the present study, the majority of lesions were located in the abdominal wall, and most abdominal wall cases had a history of previous cesarean delivery, supporting the theory of iatrogenic implantation of endometrial tissue during uterine incision and closure. However, one case occurred in the absence of prior abdominal surgery, suggesting that alternative pathogenetic mechanisms, such as lymphatic or hematogenous dissemination or coelomic metaplasia, may also contribute to the development of abdominal wall endometriosis, particularly in patients with concomitant endometrioma and deep infiltrating endometriosis.

One of the most relevant findings of our study was the association between higher CA-125 levels and muscle or fascial invasion. Although CA-125 is not specific for abdominal wall endometriosis, several studies have shown that serum CA-125 levels tend to increase with disease severity and depth of infiltration [14,15]. Previous studies have demonstrated that elevated CA-125 values are more frequently observed in advanced or deeply infiltrating endometriosis and may correlate with the extent of disease. In abdominal wall endometriosis, deeper involvement of the fascia or rectus muscle has also been associated with higher CA-125 levels and larger lesion size, and such cases may require more extensive surgical excision or mesh reconstruction [16]. In our series, mesh repair was required in two patients, both of whom had muscle and fascial invasion and lesion sizes greater than 5 cm, supporting the relationship between lesion size, depth of invasion, and surgical complexity. Overall, the CA-125 levels were significantly higher in patients with muscle or fascial invasion, suggesting that this marker may reflect lesion depth and operative difficulty rather than the presence of endometriosis alone. However, given the relatively small sample size and the absence of multivariate analysis, this association should be interpreted with caution and considered hypothesis-generating. Additionally, in our study, patients with elevated CA-125 levels were significantly older, and higher body mass index was associated with a greater number of previous cesarean sections and a higher frequency of ≥2 cesarean deliveries. Previous studies have identified prior cesarean delivery and repeated abdominal surgery as important risk factors for abdominal wall endometriosis, although data evaluating their relationship with lesion characteristics remain limited.

Clinical presentation in our series was consistent with previous reports. Menstrual cycle-related pain or swelling was present in the majority of patients and remains one of the most characteristic findings of abdominal wall endometriosis. Previous studies have reported cyclic symptoms in approximately half to two-thirds of patients, although the frequency varies between series [17,18]. In our cohort, cyclic symptoms were observed in a higher proportion of cases, emphasizing the importance of detailed clinical history in patients presenting with painful masses at previous surgical sites. However, the diagnosis may be delayed because abdominal wall endometriosis can mimic hernia, lipoma, abscess, suture granuloma, or soft tissue tumors, and some patients are initially evaluated by general surgery rather than gynecology [19].

Imaging plays an important role in the evaluation of suspected abdominal wall endometriosis. Ultrasonography is usually the first-line imaging modality because of its accessibility and low cost, and typical findings include hypoechoic or heterogeneous masses with irregular margins. Magnetic resonance imaging is particularly useful for defining lesion borders and evaluating the depth of invasion, especially when muscular or fascial involvement is suspected [20]. Computed tomography may help in selected cases to assess the relationship of the lesion to surrounding structures [21]. However, imaging findings are not always specific, and histopathological examination after surgical excision remains the gold standard for definitive diagnosis [22]. Techniques such as methylene blue injection into lesions during surgery can facilitate dissection, although this approach is not widely standardized or studied. In our series, imaging findings were used to determine lesion size, localization, and depth before surgery, and these findings were consistent with the intraoperative and histopathological results.

This study has several limitations that should be acknowledged. The retrospective design and relatively small sample size, together with the lack of multivariate analysis, may limit both the generalizability and the statistical power of the findings and should be considered when interpreting the observed associations. In addition, the single-center nature of the study may introduce selection bias. Despite these limitations, the inclusion of surgically and histopathologically confirmed cases and the comprehensive evaluation of clinical, laboratory, and operative findings provide valuable insights into this uncommon condition. Furthermore, the analysis of muscle and fascial invasion in relation to CA-125 levels highlights a potentially clinically relevant association. Future studies incorporating additional diagnostic and methodological approaches are warranted, and larger prospective multicenter studies are needed to validate these findings and to better define the role of CA-125 as a marker of disease severity.

## 5. Conclusions

Abdominal wall endometriosis should be considered in patients presenting with cyclic pain or swelling at surgical sites, particularly after cesarean delivery, although it may also occur without prior abdominal surgery. Deep muscle and fascial invasion may be associated with elevated CA-125 levels and increased CA-125 positivity, suggesting a possible association requiring further validation. These findings may support improved diagnostic awareness and more informed surgical planning.

## Figures and Tables

**Table 1:** Demographic and baseline characteristics of the study population.

Characteristics	Value
Age (years)	36 (9.00)
BMI (kg/m^2^)	27.1 (5.40)
Parity	2 (1.00)
Previous cesarean section, *n* (%)	
0	3 (10.3)
1	11 (37.9)
≥2	15 (51.8)
Previous non-cesarean abdominal surgery, *n* (%)	
Yes	10 (34.5)
No	19 (65.5)
CA-125 (U/mL)	40 (24.00)
CA-125 positivity (≥35 U/mL), *n* (%)	
Yes	20 (69)
No	9 (31)
Largest lesion size (cm)	3 (1.50)
Concomitant endometrioma, *n* (%)	
Yes	3 (10.3)
No	26 (89.7)
Location, *n* (%)	
Abdominal wall	27 (93.1)
Vaginal	2 (6.9)
Smoking history, *n* (%)	
Yes	6 (20.7)
No	23 (79.3)
Previous endometriotic lesion resection, *n* (%)	
Yes	3 (10.3)
No	26 (89.7)
Symptoms related to menstrual cycle, *n* (%)	
Yes	26 (89.7)
No	3 (10.3)

Data are presented as median (interquartile range) for continuous variables and number (percent) for categorical variables. BMI: body mass index; CA-125: cancer antigen 125; U/mL: units per milliliter.

**Table 2:** Comparison of abdominal wall endometriosis cases according to muscle/fascia invasion.

Characteristics	Muscle/Fascia Invasion Present (*n* = 17)	Muscle/Fascia Invasion Absent (*n* = 10)	*p*	OR (95% CI)
Age (years)	37 (8.00)	33 (8.00)	0.258	
BMI (kg/m^2^)	27.3 (5.90)	26.5 (1.68)	0.782	
BMI category, *n* (%) ≥25			0.406	
	10 (58.8)	8 (80)		
<25	7 (41.2)	2 (20)		
Previous cesarean section, *n*	2 (1.00)	2 (1.00)	0.893	
Previous cesarean section category, *n* (%)			1.000	
≥2	9 (52.9)	6 (60)		
<2	8 (47.1)	4 (40)		
Previous non-cesarean abdominal surgery, *n* (%)			0.692	
Yes	7 (41.2)	3 (30)		
No	10 (58.8)	7 (70)		
CA-125 (U/mL)	45 (23.00)	29 (21.50)	0.005	
CA-125 positivity (≥35 U/mL), *n* (%)			0.029	7.50 (1.09–51.5)
Yes	15 (88.2)	5 (50)		
No	2 (11.8)	5 (50)		
Largest lesion size (cm)	3 (1.00)	3.25 (1.75)	0.269	
Smoking history, *n* (%)			0.363	
Yes	5 (29.4)	1 (10)		
No	12 (70.6)	9 (90)		
Symptoms related to menstrual cycle, *n* (%)			1.000	
Yes	15 (88.2)	9 (90)		
No	2 (11.8)	1 (10)		
Mesh repair after excision, *n* (%)			0.516	
Yes	2 (11.8)	0 (0)		
No	15 (88.2)	10 (100)		

Data are presented as median (interquartile range) for continuous variables and number (percent) for categorical variables. *p* values were calculated using the Mann–Whitney U test for continuous variables and the chi-square test or Fisher’s exact test, as appropriate, for categorical variables. Analysis performed among abdominal wall endometriosis cases only (*n* = 27); vaginal endometriosis cases (*n* = 2) were not applicable for this analysis. Odds ratio (OR) with 95% confidence interval (CI) is provided for CA-125 positivity only. BMI: body mass index; CA-125: cancer antigen 125.

## Data Availability

The data supporting the findings of this study are not publicly available due to institutional data protection policies and patient confidentiality requirements. Data can be made available by the corresponding author upon reasonable request and in accordance with ethical and institutional regulations.

## Appendix A

Additional subgroup analyses were performed to explore associations between clinical variables. Patients with elevated CA-125 levels were significantly older (*p* = 0.046). Higher BMI was associated with a greater number of previous cesarean sections (*p* = 0.010) and a higher frequency of ≥2 cesarean deliveries (*p* = 0.037).

**Table A1:** Additional subgroup analyses.

Characteristics	Group 1	Group 2	*p*
**CA-125 level**	**CA-125 ≥ 35 (*n* = 20)**	**CA-125 < 35 (*n* = 7)**	
Age (years)	37.5 (8.25)	31 (8.50)	0.046
BMI (kg/m^2^)	26.8 (6.17)	27.4 (2.05)	0.599
BMI category, *n* (%) ≥25			0.363
	12 (60)	6 (85.7)	
<25	8 (40)	1 (14.3)	
Previous cesarean section, *n*	1.50 (1.00)	2 (1.00)	0.201
Previous cesarean section category, *n* (%)			0.408
≥2	10 (50)	5 (71.4)	
<2	10 (50)	2 (28.6)	
Previous non-cesarean abdominal surgery, *n* (%)			0.204
Yes	9 (45)	1 (14.3)	
No	11 (55)	6 (85.7)	
Largest lesion size (cm)	3.75 (1.00)	3.00 (1.25)	0.061
Smoking history, *n* (%)			0.633
Yes	4 (20)	2 (28.6)	
No	16 (80)	5 (71.4)	
Symptoms related to menstrual cycle, *n* (%)			1.000
Yes	18 (90)	6 (85.7)	
No	2 (10)	1 (14.3)	
Muscle/fascia invasion, *n* (%)			0.325
Yes	15 (75)	2 (28.6)	
No	5 (25)	5 (71.4)	
**BMI category**	**BMI ≥ 25 (*n* = 18)**	**BMI < 25 (*n* = 9)**	
Age (years)	36 (6.75)	36 (10.00)	0.817
Previous cesarean section, *n*	2 (0.75)	1 (0.50)	0.010
Previous cesarean section category, *n* (%)			0.037
≥2	13 (72.2)	2 (22.2)	
<2	5 (27.8)	7 (77.8)	
Previous non-cesarean abdominal surgery, *n* (%)			0.219
Yes	5 (27.8)	5 (55.6)	
No	13 (72.2)	4 (44.4)	
CA-125 (U/mL)	40 (24.00)	45 (25.00)	0.198
CA-125 positivity (≥35 U/mL), *n* (%)			0.363
Yes	12 (66.7)	8 (88.9)	
No	6 (33.3)	1 (11.1)	
Largest lesion size (cm)	3.25 (1.75)	3 (2.00)	0.510
Smoking history, *n* (%)			0.136
Yes	2 (11.1)	4 (44.4)	
No	16 (88.9)	5 (55.6)	
Symptoms related to menstrual cycle, *n* (%)			0.529
Yes	15 (83.3)	9 (100)	
No	3 (16.7)	0 (0)	
Muscle/fascia invasion, *n* (%)			0.406
Yes	10 (55.6)	7 (77.8)	
No	8 (44.4)	2 (22.2)	

Data are presented as median (interquartile range) for continuous variables and number (percent) for categorical variables. *p* values were calculated using the Mann–Whitney U test for continuous variables and the chi-square test or Fisher’s exact test, as appropriate, for categorical variables. Analysis performed among abdominal wall endometriosis cases only (*n* = 27); vaginal endometriosis cases (*n* = 2) were not applicable for this analysis. BMI: body mass index; CA-125: cancer antigen 125.
